# Natural Language Processing Versus Diagnosis Code–Based Methods for Postherpetic Neuralgia Identification: Algorithm Development and Validation

**DOI:** 10.2196/57949

**Published:** 2024-09-10

**Authors:** Chengyi Zheng, Bradley Ackerson, Sijia Qiu, Lina S Sy, Leticia I Vega Daily, Jeannie Song, Lei Qian, Yi Luo, Jennifer H Ku, Yanjun Cheng, Jun Wu, Hung Fu Tseng

**Affiliations:** 1 Department of Research & Evaluation, Kaiser Permanente Southern California, 100 S Los Robles Ave, 2nd Floor, Pasadena, CA, 91101, United States, 1 626-986-8665, 1 626-564-7872; 2 South Bay Medical Center, Kaiser Permanente Southern California, Harbor City, CA, United States; 3 Kaiser Permanente Bernard J. Tyson School of Medicine, Pasadena, CA, United States

**Keywords:** postherpetic neuralgia, herpes zoster, natural language processing, electronic health record, real-world data, artificial intelligence, development, validation, diagnosis, EHR, algorithm, EHR data, sensitivity, specificity, validation data, neuralgia, recombinant zoster vaccine

## Abstract

**Background:**

Diagnosis codes and prescription data are used in algorithms to identify postherpetic neuralgia (PHN), a debilitating complication of herpes zoster (HZ). Because of the questionable accuracy of codes and prescription data, manual chart review is sometimes used to identify PHN in electronic health records (EHRs), which can be costly and time-consuming.

**Objective:**

This study aims to develop and validate a natural language processing (NLP) algorithm for automatically identifying PHN from unstructured EHR data and to compare its performance with that of code-based methods.

**Methods:**

This retrospective study used EHR data from Kaiser Permanente Southern California, a large integrated health care system that serves over 4.8 million members. The source population included members aged ≥50 years who received an incident HZ diagnosis and accompanying antiviral prescription between 2018 and 2020 and had ≥1 encounter within 90‐180 days of the incident HZ diagnosis. The study team manually reviewed the EHR and identified PHN cases. For NLP development and validation, 500 and 800 random samples from the source population were selected, respectively. The sensitivity, specificity, positive predictive value (PPV), negative predictive value (NPV), F-score, and Matthews correlation coefficient (MCC) of NLP and the code-based methods were evaluated using chart-reviewed results as the reference standard.

**Results:**

The NLP algorithm identified PHN cases with a 90.9% sensitivity, 98.5% specificity, 82% PPV, and 99.3% NPV. The composite scores of the NLP algorithm were 0.89 (F-score) and 0.85 (MCC). The prevalences of PHN in the validation data were 6.9% (reference standard), 7.6% (NLP), and 5.4%‐13.1% (code-based). The code-based methods achieved a 52.7%‐61.8% sensitivity, 89.8%‐98.4% specificity, 27.6%‐72.1% PPV, and 96.3%‐97.1% NPV. The F-scores and MCCs ranged between 0.45 and 0.59 and between 0.32 and 0.61, respectively.

**Conclusions:**

The automated NLP-based approach identified PHN cases from the EHR with good accuracy. This method could be useful in population-based PHN research.

## Introduction

Herpes zoster (HZ) or shingles is a painful dermatomal vesicular disease that results from the reactivation of the latent varicella-zoster virus in the nerve ganglia [1]. Nearly all adults have the varicella-zoster virus dormant in their nervous system [2], and the estimated lifetime risk of HZ was approximately 30% prior to the availability of the zoster vaccine [3]. HZ usually begins with a prodromal stage of discomfort, followed by a painful, itchy rash on one unilateral dermatome that lasts 2 to 4 weeks [4]. Patients with HZ may develop postherpetic neuralgia (PHN)—dermatomal pain persisting at least 90 days after the appearance of the acute HZ rash [3,5]. PHN is the most common complication of HZ and greatly lowers patients’ quality of life [3].

Population-based studies using real-world data are cost-effective ways to address many questions about PHN [3]. However, accurately identifying PHN is difficult. Clinical trials rely on predetermined follow-up visits, which are difficult to replicate in real-world settings [6,7]. Due to time and resource constraints, prospective studies have mainly been limited to hundreds of patients with HZ and smaller numbers of PHN cases [3]. Retrospective studies of PHN have relied heavily on diagnosis codes [8-13], which lack accuracy [3,14], or manual chart review [14-16], which is costly and time-consuming. Moreover, despite the widespread use of code-based algorithms, only a few publications included PHN algorithm validation results [8,10].

Natural language processing (NLP), a subfield of artificial intelligence, has been used to identify and extract information from unstructured clinical data. We previously developed NLP methods to identify HZ ophthalmicus and HZ ophthalmicus with eye involvement, which are also common HZ complications [17,18]. In this study, we developed and validated an NLP algorithm to identify PHN. Using manual chart-reviewed results as a reference standard, we compared the performance of the NLP algorithm with that of 5 previously published code-based algorithms.

## Methods

### Setting

This study was conducted at Kaiser Permanente Southern California (KPSC), an integrated health care system with 16 hospitals and 197 medical offices that serves over 4.8 million members. The prepaid health plan incentivizes members to use services at KPSC facilities. The electronic health record (EHR) system at KPSC stores all aspects of member care, including sociodemographic characteristics, medical encounters, diagnoses, laboratory tests, pharmacy use, immunization records, membership history, and billing and claims.

### PHN Case Definition

PHN was defined as pain or discomfort consistent with the HZ episode ≥90 days after the initial HZ diagnosis; the symptoms were at the location of the initial HZ rash and were not due to other obvious causes [19-21].

### Data Sets

This study used EHR data of patients aged ≥50 years who each had an incident HZ diagnosis and associated antiviral prescription between 2018 and 2020 at KPSC. All patients had to have at least 1 year of membership prior to the index (incident HZ diagnosis) date so that comorbidities and health care use could be ascertained. Among patients with ≥1 encounter during the 90‐180 days after the incident HZ diagnosis, trained research associates reviewed their EHRs based on the PHN abstraction instructions (Multimedia Appendix 1). An infectious disease physician (BKA) reviewed all possible or unclear cases. From these reviewed cases, we randomly selected 500 cases for NLP development and 800 cases for NLP validation. Because the NLP work was done concurrently with the manual review, the development data set was collected at an earlier stage, when the reviewed cohort had a greater proportion of Asian and recombinant zoster vaccine–vaccinated patients.

### Reference Standard

Among the 800 cases in the validation data set, BKA reviewed 37 HZ cases that research associates had identified as unclear PHN cases. Because reviewers sometimes missed positive mentions of PHN, BKA rereviewed cases in the validation set where NLP results differed from reviewer results. Nine cases were corrected from negative to positive PHN. These manually reviewed results served as the reference standard for assessing the performance of PHN identification algorithms.

### NLP Algorithm Development

We developed the NLP algorithm based on our previous work [17,18,22-26]. Multimedia Appendix 2 describes the steps for preprocessing text and generating nomenclature. We created the rule-based NLP algorithm using the Linguamatics I2E software (Linguamatics, an IQVIA company). Each note was searched at different levels: section (eg, “Physical Exam,” “Assessment/Plan”), cross-sentence, intrasentence, and phrase. A distance-based relationship algorithm was applied to identify related terms based on the number of words or sentences between them. The relationship search identified the words or phrases (eg, negated, uncertain, and hypothetical statements) that modified the concepts of interest.

**Figure 1.**
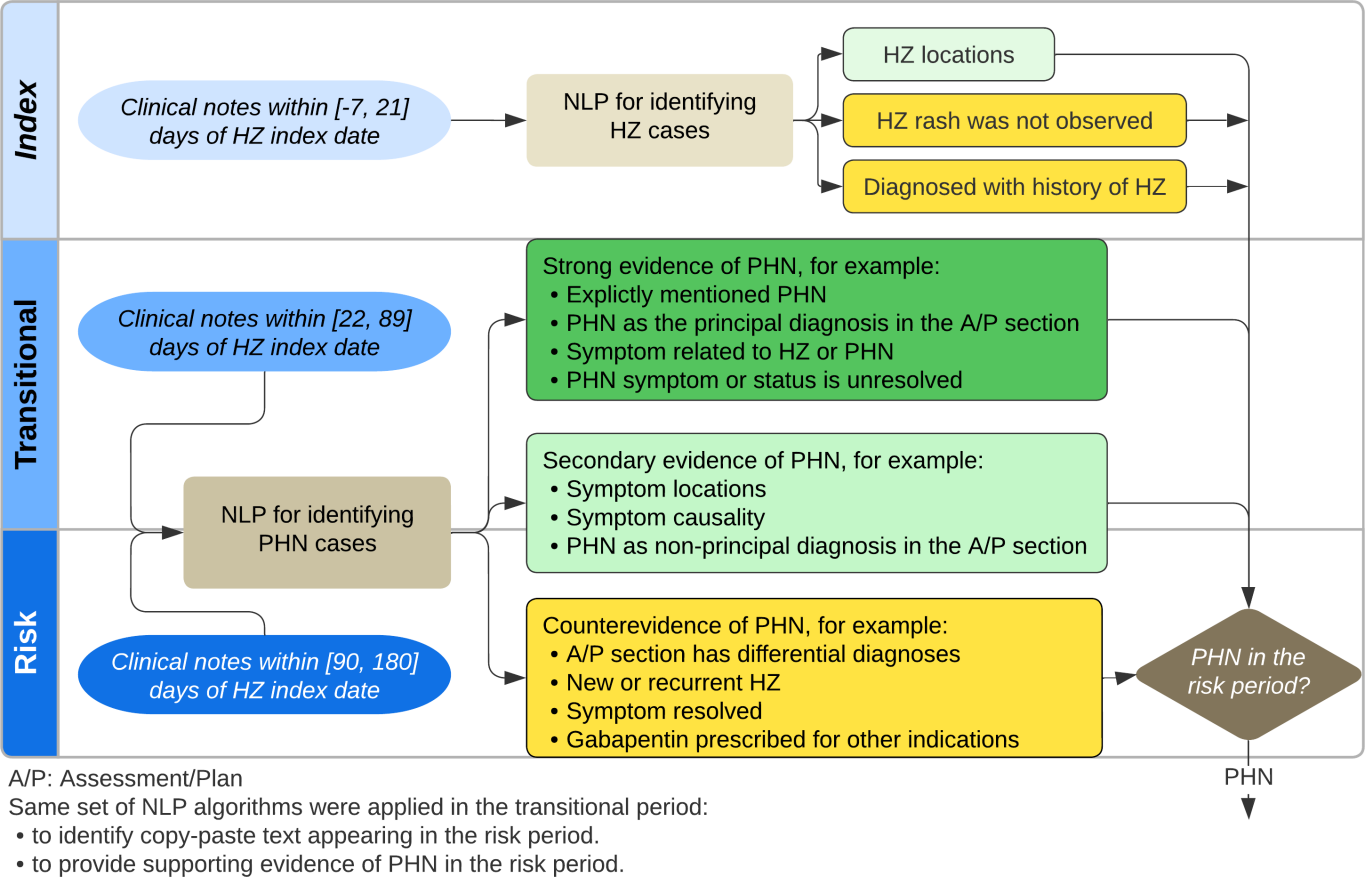
Diagram of NLP algorithm. HZ: herpes zoster; NLP: natural language processing; PHN: postherpetic neuralgia.

Figure 1 depicts an overview of the NLP algorithm. We separated the extracted clinical texts into 3 time periods: index (acute HZ) period (−7 to 21 d from incident HZ diagnosis date), transitional (subacute HZ) period (22 to 89 d), and risk (defined PHN) period (90 to 180 d). We developed search queries to identify the HZ anatomic locations in the index episode and PHN-related evidence in the transitional and risk periods. Supporting evidence of PHN included explicit mention of ongoing PHN, symptom location and causality, and PHN listed in the assessment and plan section. Counterevidence of PHN included differential diagnoses, recurrent HZ, and resolved PHN. We excluded sections and statements that may have been copied forward as historical information.

The PHN decision algorithm was implemented in Python language, which incorporated the evidence from the NLP search queries and classified each case based on decision rules. To exclude the copy-pasted results, the NLP program ran search queries on both the transitional and risk periods and compared the results to locate identical sequences of text. The algorithm considered the time sequences of identified evidence. The symptom location during the risk period was compared with the index HZ location. Because adjacent dermatomes might be difficult to distinguish clinically, symptom location during the index and risk periods had to occur in the same or surrounding dermatomes (eg, face and neck). Based on the development data set, we tested and updated the algorithm.

### Implementation of Published PHN Identification Algorithms

We selected and implemented 5 code-based PHN identification algorithms based on the variety of their algorithms, the journal category and impact factor, the publication year, the total citations, and the size of the study (Table 1). The first code-based method (C1: Yanni et al [27]) exclusively used PHN-related diagnosis codes (Multimedia Appendix 3). The remaining 4 algorithms (C2: Klompas et al [8]; C3: Klein et al [10]; C4: Forbes et al [9]; C5: Munoz-Quiles et al [11]) used additional structured data, such as diagnosis codes for HZ, neuralgia, and chronic pain; prescriptions for analgesics, antidepressants, and anticonvulsants; and clinical visit data.

**Table 1.** List of sources for selected code-based methods.

Method	Herpes zoster cases, n	Journal category	Journal (IF^a^)	Year	TC^b^
C1 [27]	21,146	General medicine	BMJ Open (2.9)	2018	67
C2 [8]	2089	General medicine	Mayo Clinic Proceeding (8.9)	2011	125
C3 [10]	62,205	Immunology	Vaccine (5.5)	2019	32
C4 [9]	119,413	Neurology	Neurology (10.1)	2016	155
C5 [11]	87,086	Infectious diseases	Journal of Infection (28.2)	2018	38

a IF: impact factor based on Journal Citation Report released in 2023.

b TC: total citations based on Google Scholar as of July 1, 2024.

### Validation and Analysis

The results generated from the various algorithms were evaluated against the chart-reviewed reference standard validation data set. We counted the numbers of true-positive (TP), false-positive (FP), true-negative (TN), and false-negative (FN) cases to calculate the performance metrics: sensitivity, specificity, positive predictive value (PPV), negative predictive value (NPV), F-score [28], and Matthews correlation coefficient (MCC) [29].

The F-score is a combination metric in machine learning and NLP research. It is defined as a weighted harmonic mean of sensitivity and PPV, where the parameter β represents the relative importance of sensitivity versus PPV.


$$F-score=\frac{(\beta^{2}+1)\;*\;PPV\;*\;sensitivity}{\beta^{2}*\;PPV\;+\;sensitivity}$$

Since a minority of patients with HZ will develop PHN and FNs and sensitivity are more important than PPV, we chose *β*=2 to favor sensitivity over PPV. The F-score’s value ranges from 0 to 1, with higher values suggesting better prediction. However, because the F-score does not include TN in its formula, MCC has been proposed as a better overall measurement than the F-score as well as the area under the receiver operating characteristic curve in binary classification [29,30]. The MCC formula considers all 4 confusion matrix categories, with values between −1 to 1, where ±1 denotes perfect agreement or disagreement between actuals and predictions, and 0 indicates randomness.


$$MCC=\frac{TP*TN-FP*FN}{\sqrt{\left(TP+FP)*(TP+FN)*(TN+FP)*(TN+FN\right)}}$$

The prevalence proportion of PHN was calculated as the number of identified PHN cases per 100 cases of HZ.

### Ethical Considerations

The KPSC institutional review board approved this study (institutional review board number: 12270). A waiver of informed consent was granted for this study because this was a data-only minimal-risk study.

## Results

### Study Population

The characteristics of the study population are presented in Table 2. The mean (SD) ages of the development and validation data sets were 69.5 (9.1) and 70.0 (9.8) years, respectively, with 329 (65.8%) and 542 (67.8%) being female. The development data set had a higher proportion of Asian (177/500, 35.4% vs 110/796, 13.8%) and recombinant zoster vaccine–vaccinated patients (61/500, 12.2% vs 28/800, 3.5%). There were no significant differences between the development and validation data sets in terms of clinical visits and comorbidities prior to the index date. In the development and validation data sets, approximately one-third of the patients had diabetes (159/500, 31.8% and 262/796, 32.8%, respectively), while less than one-quarter had chronic pulmonary disease (88/500, 17.6% and 163/800, 20.4%) and depression (103/500, 20.6% and 189/800, 23.6%). The development data had a higher proportion of patients with cancer as compared with the validation data (52/500, 10.4% vs 54/800, 6.8%).

**Table 2.** Characteristics of patients in the development and validation data sets.

Characteristics		Patients		*P* value^a^
		Development (n=500)	Validation (n=800)	
Age (year), mean (SD)		69.5 (9.1)	70.0 (9.8)	.47
**Age group (years), n (%)**				.52
	50‐59	70 (14.0)	103 (12.9)	
	60‐69	172 (34.4)	282 (35.3)	
	70‐79	187 (37.4)	280 (35.0)	
	≥80	71 (14.2)	135 (16.9)	
**Sex, n (%)**				.47
	Female	329 (65.8)	542 (67.8)	
	Male	171 (34.2)	258 (32.3)	
**Race or ethnicity, n (%)**				<.01
	Non-Hispanic White	198 (39.6)	424 (53.0)	
	Hispanic	102 (20.4)	213 (26.6)	
	Asian/Pacific Islanders	177 (35.4)	110 (13.8)	
	Non-Hispanic Black	18 (3.6)	41 (5.1)	
	Other/Multiple/Unknown	5 (1.0)	12 (1.5)	
**Number of outpatient/digital visits 6 months before HZ**^**b**^ **diagnosis date, n (%)**				.91
	0‐1	68 (13.6)	106 (13.3)	
	2‐5	168 (33.6)	262 (32.8)	
	≥6	264 (52.8)	432 (54.0)	
**Number of emergency department visits 6 months before HZ diagnosis date, n (%)**				.08
	0	420 (84.0)	641 (80.1)	
	≥1	80 (16.0)	159 (19.9)	
**Number of hospitalizations 6 months before HZ diagnosis date, n (%)**				.45
	0	475 (95.0)	752 (94.0)	
	≥1	25 (5.0)	48 (6.0)	
**Comorbidity 1 year before HZ diagnosis date, n (%)**				
	Allergic rhinitis	38 (7.6)	51 (6.4)	.39
	Asthma	41 (8.2)	82 (10.3)	.22
	Atopic dermatitis	5 (1.0)	9 (1.1)	.83
	Cancer	52 (10.4)	54 (6.8)	.02
	Chronic pulmonary disease	88 (17.6)	163 (20.4)	.22
	Depression	103 (20.6)	189 (23.6)	.20
	Diabetes	159 (31.8)	262 (32.8)	.72
	Epilepsy and recurrent seizures	8 (1.6)	8 (1.0)	.34
	Heart failure	24 (4.8)	55 (6.9)	.13
	Rheumatoid arthritis	25 (5.0)	38 (4.8)	.84
	Systemic lupus erythematosus	6 (1.2)	6 (0.8)	.41
**Recombinant zoster vaccine, n (%)**				<.01
	Unvaccinated	439 (87.8)	772 (96.5)	
	1-dose vaccinated	32 (6.4)	9 (1.1)	
	Fully (2-dose) vaccinated	29 (5.8)	19 (2.4)	

a *χ*2 test was used for categorical variables, and Wilcoxon test was used for continuous variables.

b HZ: herpes zoster.

### Validation Data Set

In the validation data set, the numbers of clinical notes in the index, transitional, and risk periods were 12,158, 14,446, and 18,895, respectively. The percentages of HZ- or PHN-relevant notes were 26.2%, 8.2%, and 3.2%, respectively for the index, transitional, and risk periods. Most of the HZ index visits occurred in primary care, urgent care, emergency departments, and hospital settings (Multimedia Appendix 4). After the index period, HZ-related mentions were much less frequently documented in urgent care visit notes, but more frequently documented in specialist visit notes (41 specialties).

### Application of NLP on Validation Data Set

Out of the 800 patients in the validation data set, the NLP algorithm identified 796 patients with HZ who had at least 1 note with HZ- or PHN-related terms in the index period. Among the 4 remaining patients, 2 patients had their index HZ diagnosed outside KPSC and had no follow-up visits in the index period. For the remaining 2 patients, HZ-related symptoms were documented, but no mention of HZ or PHN was made in the clinical notes. Among these 796 patients, the NLP algorithm identified the HZ anatomic location for 751 (94.3%) patients, and among them, 611 (81.3%) had laterality information (Multimedia Appendix 5). In the transitional and risk periods, the NLP algorithm identified positive mentions of any pain or discomfort in 370 (46.3%) and 425 (53.1%) patients, respectively.

### Validation Results

In the validation data set, the NLP algorithm achieved a 90.9% sensitivity, 98.5% specificity, 82% PPV, and 99.3% NPV (Table 3). The composite scores of the NLP algorithm were 0.89 (F-score) and 0.85 (MCC). Of the 800 patients in the validation data set, 55 (6.9%) were chart-confirmed as PHN. The prevalence proportion of PHN identified by the NLP algorithm was 7.6%.

**Table 3.** Performance characteristics of natural language processing and code-based methods for identifying postherpetic neuralgia as compared with chart-confirmed reference standard.

Method	PHN^a^ (%)	TP^b^	TN^c^	FN^d^	FP^e^	Sensitivity (%)	Specificity (%)	PPV^f^ (%)	NPV^g^ (%)	F-score	MCC^h^
NLP^i^	7.6	50	734	5	11	90.9	98.5	82.0	99.3	0.89	0.85
C1	5.4	31	733	24	12	56.4	98.4	72.1	96.8	0.59	0.61
C2	10.9	34	692	21	53	61.8	92.9	39.1	97.1	0.55	0.44
C3	5.5	31	732	24	13	56.4	98.3	70.5	96.8	0.59	0.61
C4	13.1	29	669	26	76	52.7	89.8	27.6	96.3	0.45	0.32
C5	9.3	31	702	24	43	56.4	94.2	41.9	96.7	0.53	0.44

a PHN: postherpetic neuralgia.

b TP: true-positive.

c TN: true-negative.

d FN: false-negative.

e FP: false-positive.

f PPV: positive predictive value.

g NPV: negative predictive value.

h MCC: Matthews correlation coefficient.

i NLP: natural language processing.

### Error Analysis of NLP Validation Results

Error analysis of the FN and FP cases is presented in Table 4. Some of the NLP-related errors were caused by the selection of data sources. For 2 FN cases, NLP incorrectly classified them as PHN negative when statements were found indicating HZ-associated pain had resolved even though additional evidence showed the patients still had other PHN-related symptoms. The FP cases were caused by copied-and-pasted text, incorrect causality attribution of symptoms, misclassified recurrent HZ cases as PHN, and unclear clinical documentation.

**Table 4.** Error analysis of natural language processing false-negatives and false-positives.

Type of NLP^a^ error		Description	Number of cases
**False-negative**			5
	EHR^b^ data source	Case 1: We did not include one free text table (formatted messages) from the Epic EHR. Case 2: PHN^c^ was mentioned in a clinical note from the hematology department, which was excluded from NLP processing.	2
	Unclear documentation	HZ^d^ or PHN was not stated in the clinical note, which was required by NLP to reduce false-positive hits.	1
	Symptom	While the patient stated that HZ-associated pain had resolved, documents also indicated that the patient still had other PHN-related symptoms (prickling sensation and itchy).	2
**False-positive**			11
	EHR data source	We included Epic’s SmartData elements, which lacked specificity for PHN identification.	2
	Unclear documentation	In 2 cases, the text was copied from the clinical notes in the index period. The NLP copy-and-paste detection algorithm was only applied to the clinical notes in the transitional period. In another 2 cases, PHN and PHN-related medications were listed in the assessment and plan sections. However, it was unclear whether the patient had ongoing symptoms.	4
	Acute HZ	NLP misclassified 2 acute HZ cases that occurred in the risk period as PHN.	2
	Causality	Case 1: Pain thought to be due to chalazion based on information in follow-up visits. Case 2: PHN was listed in the assessment section and tramadol and gabapentin were listed in the plan section. However, the medications were likely for lumbosacral radiculopathy.	2
	Symptom	The patient reported generalized symptoms (nausea) since HZ, but there was no mention of concomitant sensory changes such as pain, thus the case did not meet our PHN definition.	1

a NLP: natural language processing.

b EHR: electronic health record.

c PHN: postherpetic neuralgia.

d HZ: herpes zoster.

### Code-Based Methods

The prevalence proportions of PHN identified by code-based methods ranged from 5.4% to 13.1%. The code-based methods achieved a 52.7%‐61.8% sensitivity, 89.8%‐98.4% specificity, 27.6%‐72.1% PPV, and 96.3%‐97.1% NPV. The F-scores and MCCs ranged between 0.45 and 0.59 and between 0.32 and 0.61, respectively. The more sophisticated algorithms were no better than the PHN diagnosis code–only method as measured by the F-score or MCC. Although each component of the code-based methods identified PHN cases, most of them did not contribute to identifying additional true PHN cases beyond those identified by PHN diagnosis codes, and those that did have much lower PPVs (C4.3: 10.3%, C4.2: 26.1% and C2.2: 37.7%) than the PHN diagnosis code–only method (C1, PPV 72.1%) (Table 5). We re-reviewed all FP cases from code-based methods C1 and C3 and randomly sampled the remaining FP cases from approaches C2, C4, and C5. Among the 20 reviewed FP cases, we found that none were true PHN cases.

**Table 5.** Postherpetic neuralgia cases identified by code-based methods.

Method			PHN^a^ diagnosis code used	PHN, n (%)	TP^b^ (PPV^c^), n (%)	Supplementary TP^d^
C1^e^			✓	43 (5.4)^f^	31 (72.1)^f^	—^g^
**C2**			✓	87 (10.9)^f^	34 (39.1)^f^	3^f^
	C2.1		✓	43 (5.4)	31 (72.1)	—
	C2.2			69 (8.6)	26 (37.7)	3
	C2.3			4 (0.5)	1 (25.0)	—
**C3**			✓	44 (5.5)^f^	31 (70.5)^f^	—
	C3.1		✓	24 (3)	18 (75.0)	—
	C3.2		✓	7 (0.9)	7 (100.0)	—
	C3.3		✓	41 (5.1)	29 (70.7)	—
	C3.4		✓	23 (2.9)	17 (73.9)	—
**C4**			✓	105 (13.1)^f^	29 (27.6)^f^	2^f^
	C4.1		✓	36 (4.5)	26 (72.2)	—
	**C4.2**			69 (8.6)	18 (26.1)	2
		C4.2.1		7 (0.9)	6 (85.7)	—
		C4.2.2		2 (0.3)	1 (50.0)	—
		C4.2.3		64 (8.0)	16 (25.0)	1
		C4.2.4		2 (0.3)	1 (50.0)	1
	**C4.3**			29 (3.6)	3 (10.3)	2
		C4.3.1		25 (3.1)	1 (4.0)	1
		C4.3.2		2 (0.3)	1 (50.0)	1
		C4.3.3		2 (0.3)	1 (50.0)	—
**C5**			✓	74 (9.3)^f^	31 (41.9)^f^	—
	C5.1		✓	43 (5.4)	31 (72.1)	—
	C5.2			18 (2.3)	14 (77.8)	—
	C5.3			34 (4.3)	2 (5.9)	—

a PHN: postherpetic neuralgia.

b TP: true-positive.

c PPV: positive predictive value.

d Supplementary contributions to the number of correctly identified positive cases, apart from method C1.

e Method C1 only used PHN diagnosis codes.

f Overall performance.

g Not applicable.

## Discussion

### Principal Findings

We developed and validated NLP algorithms to identify PHN using various clinical data sources from EHRs. Compared with the chart-reviewed reference standard, the NLP algorithms showed high accuracy. This study demonstrates the feasibility of population-based PHN studies using EHR data with an automated method.

Using manual review to identify PHN cases is often infeasible for population-based research because a large volume of clinical notes would need to be reviewed. In contrast, the size of the study population and length of follow-up have little impact on running the NLP algorithm. Moreover, our NLP algorithm can readily capture PHN at varied time intervals, providing an efficient method to assess the long-term impact of PHN and compare results with studies using different PHN risk windows. Furthermore, studies can use NLP alone or with manual review confirmation. For example, a manual review of the NLP-positive cases (n=61) could increase the specificity and PPV to 100% and improve the F-score from 0.89 to 0.93 and MCC from 0.85 to 0.95; this is more efficient than a manual review of all 800 HZ cases.

Implementing NLP on EHR data presents challenges. In this study, data sources accounted for one-quarter of NLP errors (2 FNs and 2 FPs). First, clinical data were stored in a variety of locations within our institution’s complex EHR system, which contains over 900,000 database tables. It is often difficult to locate the database table storing the data displayed in the EHR user interface. One FN case resulted from not including a previously unknown table. Second, selecting data sources for NLP processing is often a tradeoff. One FN and 2 FP cases resulted from including or excluding certain data sources. EHRs have also made it easy to create lengthy and bloated notes [31,32]. According to recent research, over half of clinical note content is duplicated or copied from earlier notes [32-34]. Clinicians may copy from prior visit notes to improve recall and clinical reasoning [35]. However, these replicated contents may lack temporal or contextual information, making them difficult to identify manually and challenging for NLP.

Because PHN-related symptoms such as pain and discomfort are common in a variety of medical conditions with numerous plausible causes, identifying PHN necessitates integrating the NLP-identified PHN symptoms with their associated anatomic location, temporality, and causality. These elements, however, are not always explicitly stated in clinical documents. About half of the NLP FP cases were from incorrectly attributing the complaint or treatment to PHN. These FP cases were partially explained by the NLP algorithm’s preference for sensitivity over specificity.

Another popular method of PHN identification is using coded data from administrative claims or EHR, which could include a large sample size at a low cost. However, many of the code-based PHN identification algorithms have not been validated [3]. We implemented and validated 5 code-based algorithms, including 1 that solely uses PHN diagnosis codes (C1) and 2 that had previously been validated (C2 and C3). To maximize their sensitivity, algorithms C2-C5 used the “OR” statement to combine various criteria. The downside of using the “OR” logic is the loss of PPV. Algorithms C2-C5 all had worse PPV than the diagnosis codes–only algorithm (C1). However, in our study, the sensitivity of these algorithms ranged from 53% to 62%, with only C2 outperforming C1 (62% vs 56%). Algorithms C2-C5 had lower PPVs (28%‐71%) than C1 (72%). With such limited sensitivities, these algorithms may miss roughly half of the PHN cases. In our study, aside from the PHN diagnosis codes, the other diagnosis codes and prescription data had little impact on true case identification, instead adding complexity and increasing FPs.

Studies have used the similarity of the PHN proportions to construct the validity of their case-finding algorithms [8-11]. Administrative database studies reported PHN (pain persisting for ≥90 days) prevalences of 3%‐14% (Multimedia Appendix 6) [3], which are comparable to the 5.4%‐13.1% prevalences of the code-based approaches in our study. The broad range of prevalences identified in previous code-based studies could be caused by variations in study design, population, and data source [3]. However, the code-based approaches in this study had the same population and data source. Only the variation in algorithms could cause such a wide disparity.

We expanded the validations conducted for the 2 previously validated algorithms, which were performed on EHR data. The C2 (Klompas et al [8]) algorithm was only validated with the 30-day definition in the original study, and it had 86% sensitivity and 78% PPV. In our study, algorithm C2 with the 90-day definition had notably lower sensitivity (62%) and PPV (39%). One main contributor to the variability in performance is the difference in the temporal criteria. According to Yawn [36], up to 75% of pain present at 30 days disappears at 90 days, and the prevalence of PHN decreased by sixfold when the definition was changed from 30 days to 90 days. As prevalence decreases, so do the sensitivity and PPV [37,38]. The same trends were also reported in the original C2 paper; the PPVs for different PHN search criteria using the 30-day definition (29%‐95%) were nearly double that of using the 90-day definition (15%‐52%). The discrepancy in C2 algorithm performance between the original study and this study could be further explained by the differences in case definition. Our case definition for PHN is based on persistent PHN-related symptoms and causal attribution, not diagnosis code or medication. Algorithm C2 used ongoing symptoms or renewal of medication for HZ. The use of medications to identify PHN has some drawbacks, as PHN-related medications have a wide range of indications. For example, gabapentin, a first-line therapy for PHN, has over 20 approved and off-label uses [39]. Furthermore, prescriptions can be refilled in the absence of active PHN symptoms for various non-PHN disorders.

The original C3 (Klein et al [10]) algorithm was only validated on potential PHN cases identified by its 4 component criteria, rather than randomly selected HZ cases; only PPVs were reported. In this study, the 4 criteria of the C3 algorithm had PPVs ranging from 71% to 100%, which is consistent with the previous study’s findings (PPVs ranging from 73% to 96%). The C3 algorithm was one of the best-performing code-based algorithms based on F-score and MCC. However, its low sensitivity (56%) and PPV (71%) indicate considerable misclassification. The lower overall PPV is partly due to the “OR” logic of the 4 criteria. Because Klein et al [10] did not describe the case definition or chart review rules, we were unable to assess their impact on the performance differences between the original C3 study and this study.

The substantial misclassification of coded methods as observed in this study could have a substantial impact on measuring incidence, identifying risk factors, and assessing vaccine effectiveness. Code-based method studies (C4 and C5) had identified depression, diabetes mellitus, heart failure, and chronic obstructive pulmonary disease as risk factors for PHN. It is conceivable that the link between depression and PHN is caused by using anticonvulsants and tricyclic antidepressants to identify PHN. The inclusion of prescriptions for pain medications and chronic pain codes may contribute to the association of diabetes mellitus [40], heart failure [41], and chronic obstructive pulmonary disease [42] with PHN.

### Study Strengths and Limitations

This study was conducted within a large integrated health care system with comprehensive EHRs. Because the health plan provides strong incentives for members to use its facilities, clinical documentation is expected to be more detailed. We developed NLP algorithms to identify PHN from various unstructured data sources within EHRs, such as clinical notes, which contain a wealth of information but differ greatly in structure, content, and quality. The algorithms were highly accurate, as evidenced by our validation. Compared with studies based on self-reported pain scores collected through surveys, EHR-based studies measure the health care burden of PHN, which is more clinically relevant. This study also has limitations. The reference standard relied on the review of EHRs which could be erroneous and incomplete [14]. Moreover, rereviewing cases in the validation set where NLP results differed from research associates’ results may result in bias in favor of higher performance of the NLP algorithm. On the other hand, reconciling discrepant results improved the quality of the reference standard. Additionally, diagnosis codes, prescriptions, clinical documentation language, and style can differ between institutions and physicians. Our NLP method may perform differently in other test data sets.

### Conclusions

PHN-related diagnosis codes have low sensitivity for identifying PHN cases. Additional diagnosis codes and prescription data did little to improve sensitivity while significantly lowering the PPV. Using clinical text from the EHR, the NLP-based method identified PHN cases with high accuracy. Our NLP method can be used in EHR-based studies to identify PHN risk factors and evaluate the effectiveness of vaccinations and treatments against PHN.

## Supplementary material

10.2196/57949 Multimedia Appendix 1 Postherpetic neuralgia abstraction decision rules.

10.2196/57949 Multimedia Appendix 2 Additional details on natural language processing algorithm development.

10.2196/57949 Multimedia Appendix 3 Code-based methods.

10.2196/57949 Multimedia Appendix 4 The proportion of herpes zoster– or postherpetic neuralgia–related notes by department/specialty.

10.2196/57949 Multimedia Appendix 5 Number and percentage of herpes zoster locations as identified by natural language processing.

10.2196/57949 Multimedia Appendix 6 Reported postherpetic neuralgia rates in previous administrative database studies.

## Abbreviations

EHR: electronic health record
FN: false-negative
FP: false-positive
HZ: herpes zoster
KPSC: Kaiser Permanente Southern California
MCC: Matthews correlation coefficient
NLP: natural language processing
NPV: negative predictive value
PHN: postherpetic neuralgia
PPV: positive predictive value
TN: true-negative
TP: true-positive
