# Supplementary material for: Natural Language Processing Versus Diagnosis Code–Based Methods for Postherpetic Neuralgia Identification: Algorithm Development and Validation
Source: JMIR Med Inform. 2024 Sep 10;12:e57949. doi: 10.2196/57949 (PMC11407135; doi:10.2196/57949)
Supplement: Multimedia Appendix 1 [file medinform-v12-e57949-s001.docx]

**Appendix 1. PHN Abstraction Decision Rules**

PHN:

Pain/discomfort in the 90-180 days after the initial HZ diagnosis is consistent with the location of the initial HZ rash and not clearly related to another cause; the provider correctly attributes pain/discomfort to PHN.

Not PHN:

No mention of pain/discomfort in the 90-180 days after the initial HZ diagnosis; pain/discomfort is related to other causes; pain/discomfort is clearly not in the location of the HZ rash.

Possible PHN/unable to determine:

The provider suspects PHN but it is not confirmed; unclear whether the pain/discomfort in the 90-180 days after the initial HZ diagnosis is in the location of the HZ rash; unclear whether the pain/discomfort may be due to another cause.

“Possible PHN/unable to determine” cases were referred to an infectious disease physician for a final PHN/not PHN determination.
