# Supplementary material for: Natural Language Processing Versus Diagnosis Code–Based Methods for Postherpetic Neuralgia Identification: Algorithm Development and Validation
Source: JMIR Med Inform. 2024 Sep 10;12:e57949. doi: 10.2196/57949 (PMC11407135; doi:10.2196/57949)
Supplement: Multimedia Appendix 2 [file medinform-v12-e57949-s002.docx]

**Appendix 2. Additional Details on Natural Language Processing (NLP) Algorithm Development**

**Pre-processing of the free text data**

The retrieved free text data first underwent pre-processing steps which included encoding normalization, section detection, and sentence separation. The pre-processed free text data were formatted as Extensible Markup Language (XML) and fed into Linguamatics I2E. I2E performed tokenization (that is, segmenting text into linguistic units such as words and punctuation) and annotations for matched concepts and general linguistic entities (e.g., lexical chunks like nouns or verb phrases).

**Terminology development**

We first created study-specific terminologies based on the Unified Medical Language System. We expanded the derived terminologies by including misspellings, morphological variants, and synonyms through synonym discovery methods. We trained subword embedding models based on the training data using fastText^a^. The trained model was used to identify similar terms based on their contexts.

^a^: Bojanowski P, Grave E, Joulin A, Mikolov T. Enriching word vectors with subword information. *Transactions of the Association for Computational Linguistics*. 2017;5:135-146.
