# Supplementary material for: Natural Language Processing Versus Diagnosis Code–Based Methods for Postherpetic Neuralgia Identification: Algorithm Development and Validation
Source: JMIR Med Inform. 2024 Sep 10;12:e57949. doi: 10.2196/57949 (PMC11407135; doi:10.2196/57949)
Supplement: Multimedia Appendix 3 [file medinform-v12-e57949-s003.docx]

**Appendix 3. Code-Based Methods**

**Code-Based Method C1**

Any PHN diagnosis codes (Table S3) given during the 90-180-day risk period.

**Table S3. Diagnosis Codes for Identification of PHN**

| **ICD-10-CM** | **Description** |
| --- | --- |
| B02.2 | Zoster with other nervous system involvement |
| B02.21 | Postherpetic geniculate ganglionitis |
| B02.22 | Postherpetic trigeminal neuralgia |
| B02.23 | Postherpetic polyneuropathy |
| B02.24 | Postherpetic myelitis |
| B02.29 | Other postherpetic nervous system involvement |

Abbreviations: ICD-10-CM, International Classification of Diseases, Tenth Revision, Clinical Modification.

**PHN cases defined by method C1:** Any code in Table S3.

**Code-Based Method C2**

**C2.1:**

an ICD code for HZ with nervous system complications (B02.2x) within 90-180 days after the HZ index date

**C2.2:**

At least one additional visit with an ICD code for HZ (B02.x) ≥ 21 days after the HZ index date **AND**

(a new prescription for an analgesic 0 to 60 days after HZ codes in patients without an analgesic prescription in the preceding year,

**OR** a new prescription for an antidepressant 0 to 60 days after HZ codes in patients without an antidepressant prescription in the preceding year,

**OR** a new prescription for an anticonvulsant 0 to 60 days after HZ codes in patients without an anticonvulsant prescription in the preceding 2 years)

**C2.3:**

an ICD code (M54.10, M79.2) for neuralgia within 90-180 days after the HZ index date

**PHN cases defined by method C2:** C2.1 OR C2.2 OR C2.3

**Code-Based Method C3**

**C3.1:** Visit with PHN diagnosis^a^ and prescription with PHN diagnosis within 90-180 days post index HZ event.

**C3.2:** Visit with primary PHN diagnosis (no prescription with PHN diagnosis) within 90-180 days post index HZ event.

**C3.3:** Visit with secondary PHN diagnosis (no prescription with PHN diagnosis) within 90-180 days post index HZ event.

**C3.4:** Prescription with PHN diagnosis (no visit with PHN diagnosis) within 90-180 days post index HZ event.

**PHN cases defined by method C3:** C3.1 OR C3.2 OR C3.3 OR C3.4

^a^ **PHN diagnosis:**

all ICD-10 codes B02.22, B02.23; and ICD-10 code B02.29 where the internal KP diagnosis text indicated PHN associated with a clinical encounter (clinic visit, emergency room visit, or hospital stay)

**Code-Based Method C4**

PHN was identified using a modified algorithm based on the method C2. No validation was performed for this algorithm.

Like the original paper, we excluded patients who had a history (12 months to 2 weeks prior) of epilepsy or neuropathy prior to HZ diagnosis.

ICD-10 codes for epilepsy: G40.x

ICD-10 codes for neuropathy: G50.0, G60-G65, M79.2

**C4.1 (Definite):**

PHN code (B02.2x) within the defined period (90–180 days post HZ).

Note: In the original paper, PHN codes included both Read and ICD codes. In this study, we only used ICD codes.

**C4.2 (Probable)**:

**C4.2.1:** ICD code for HZ (B02.x) and PHN prescription^a^ on the same day (90–180 days post HZ)

**C4.2.2:** First ever non-specific neuralgia code (M79.2) 90-180 days after HZ, with no previous neuralgia code (M79.2) in the 365-14 days before the date of HZ diagnosis

**C4.2.3:** New anticonvulsant / capsaicin cream / lidocaine patch prescription, 90-180 days after HZ diagnosis

**C4.2.4:** New tricyclic antidepressant (TCA)^b^ prescription, 90-180 days after HZ with no other indication present AND a previous^c^ TCA prescription for HZ

**C4.2 =** C4.2.1 OR C4.2.2 OR C4.2.3 OR C4.2.4

**C4.3 (Possible)**:

**C4.3.1:** New strong painkiller^d^ prescription, 90-180 days after HZ with no other indication for the painkiller on the day of the prescription AND a previous^c^ strong painkiller prescription for HZ

**C4.3.2:** New TCA prescription, 90-180 days after HZ with no other indication for the TCA on the day of the prescription

**C4.3.3:** Nonspecific neuropathic pain code (G62.9), 90-180 days after HZ

**C4.3 =** C4.3.1 OR C4.3.2 OR C4.3.3

**PHN cases defined by method C4:** C4.1 OR C4.2 OR C4.3

**Notes**

^a^ PHN prescription included anticonvulsants, tricyclic antidepressants (TCA), capsaicin cream, or lidocaine patch.

New medications were defined as no previous prescription of the same drug class 12 months to two weeks before HZ diagnosis.

^b^ Tricyclic antidepressants (TCA)

Amitriptyline

Amoxapine

Clomipramine

Desipramine

Dosulepin

Doxepin

Imipramine

Lofepramine

Nortriptyline

Protriptyline

Trimipramine

^c^ A previous prescription for HZ is defined as HZ/PHN code and prescription (TCA or strong painkiller) on the same day, 0-89 days after HZ.

^d^ Strong painkiller is not listed in the original paper. We used the opioids listed below:

Buprenorphine, Butorphanol, Codeine, Fentanyl, Hydrocodone, Hydromorphone, Levorphanol, Meperidine, Methadone, Morphine, Oxycodone, Oxymorphone, Pentazocine, Propoxyphene, Tapentadol, Tramadol

**ICD-10 codes for other indications for TCA:**

Depression: F32.x, F33.x, F34.1, F41.8, F43.21, F43.23, F06.31, F06.32, F25.1

Obsessive-compulsive disorder (OCD): F42.x

Fibromyalgia: M79.7

Nocturnal enuresis: N39.44

**ICD-10 codes for other indications for a strong painkiller:**

Headache: G44.009, G44.029, G44.209

Hereditary and idiopathic neuropathy: G60.9

Unclassified pain: G89.x

Joint pain: M25.5x

Dorsalgia: M54.x

Myalgia or muscle pain: M79.1x

Fibromyalgia: M79.7

**Code-Based Method C5**

**C5.1:** the presence of an ICD code for HZ followed by an ICD code indicative of PHN (B02.22, B02.23, B02.29) within the defined period (90–180 days post HZ)

**C5.2**: the presence of an ICD code for HZ (B02.x) followed by a prescription consistent with PHN (listed below) during the defined period (90–180 days post HZ)

Medication registered for HZ and PHN:

- - Direct-acting antivirals
    - Aciclovir
    - Famciclovir
    - Valaciclovir
  - Opioids
    - Tramadol
  - Antiepileptics
    - Phenytoin
    - Carbamazepine
    - Gabapentin
    - Pregabalin
  - Antidepressant
    - Amitriptyline
    - Nortriptyline
    - Imipramine
    - Desipramine
  - Local anesthetics
    - Capsaicin
    - Lidocaine

**C5.3**: the presence of an ICD code for HZ (B02.x) followed by an ICD code for chronic pain (G89.29) during the defined period (90–180 days post HZ)

**PHN cases defined by method C5:** C5.1 OR C5.2 OR C5.3
