# Supplementary material for: Natural Language Processing Versus Diagnosis Code–Based Methods for Postherpetic Neuralgia Identification: Algorithm Development and Validation
Source: JMIR Med Inform. 2024 Sep 10;12:e57949. doi: 10.2196/57949 (PMC11407135; doi:10.2196/57949)
Supplement: Multimedia Appendix 4 [file medinform-v12-e57949-s004.docx]

**Appendix 4. The Proportion of HZ- or PHN-related Notes by Department/Specialty**


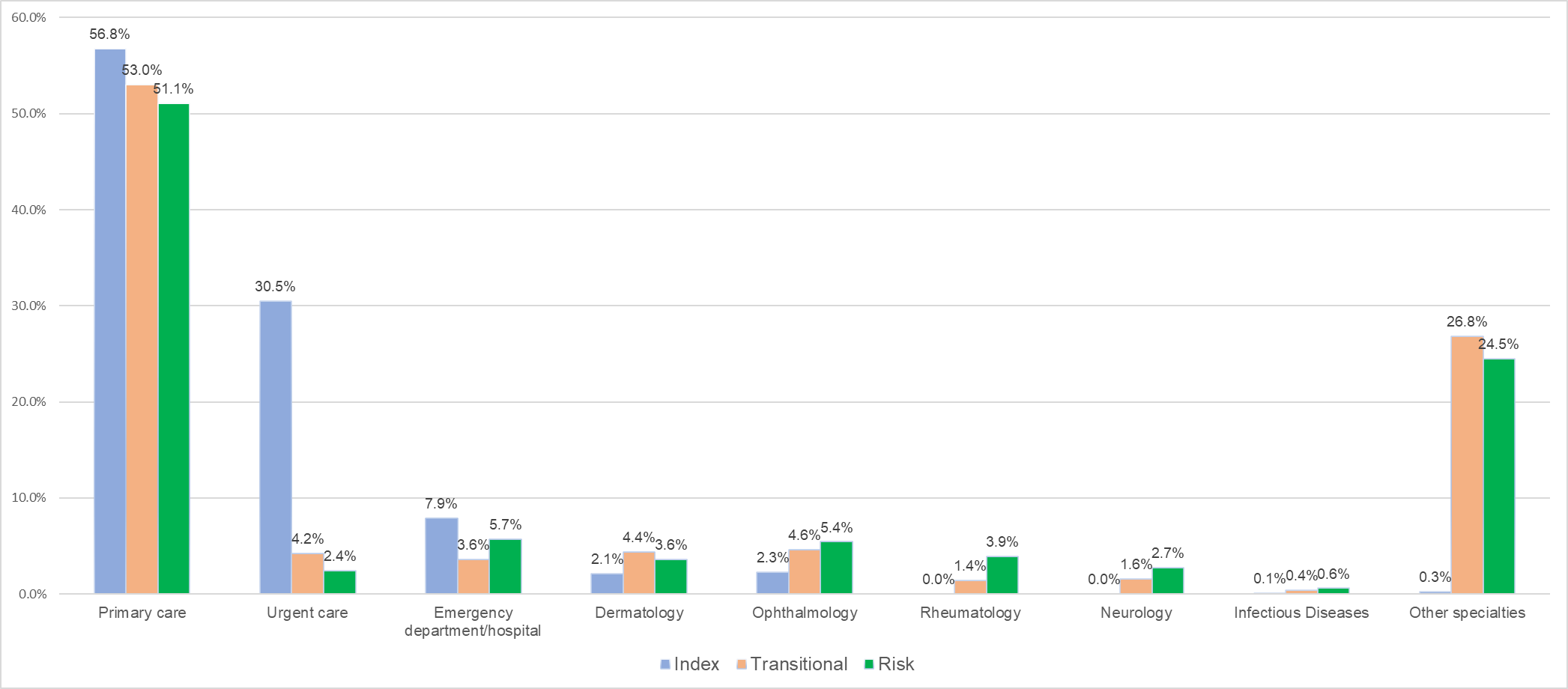
We searched for notes mentioning HZ (herpes zoster) or PHN (postherpetic neuralgia) that were not in excluded sections such as ‘Past Medical History’.

The index date was the date of HZ diagnosis.

The index period was [-7, 21] days; the transitional period was [22, 89] days, and the risk period was [90, 180] days.

**Index period**: During the index period, 796 patients had at least one note with HZ or PHN-related terms. For each patient, we selected the note nearest to the index date and identified the department/specialty associated with the acute HZ event.

**Transitional and risk periods**: Compared to the index period, the transitional and risk periods were more likely to have multiple visits. There were 500 and 331 unique combinations of patient and specialty in the transitional and risk periods, respectively.
