# Supplementary material for: Natural Language Processing Versus Diagnosis Code–Based Methods for Postherpetic Neuralgia Identification: Algorithm Development and Validation
Source: JMIR Med Inform. 2024 Sep 10;12:e57949. doi: 10.2196/57949 (PMC11407135; doi:10.2196/57949)
Supplement: Multimedia Appendix 5 [file medinform-v12-e57949-s005.docx]

**Appendix 5. Number and Percentage of HZ Locations as Identified by NLP**

| **NLP-identified HZ location** | **n** | **%** |
| --- | --- | --- |
| **Head** | 44 | 5.9 |
| Scalp | 35 | 4.7 |
| Face | 42 | 5.6 |
| V1 | 96 | 12.8 |
| V2 | 9 | 1.2 |
| V3 | 40 | 5.3 |
| Mouth | 14 | 1.9 |
| Cheek | 12 | 1.6 |
| **Neck** | 50 | 6.7 |
| **Axilla** | 20 | 2.7 |
| **Upper extremity** | 58 | 7.7 |
| Shoulder | 36 | 4.8 |
| Arm | 3 | 0.4 |
| Hand | 18 | 2.4 |
| **Torso** | 298 | 39.7 |
| Chest | 136 | 18.1 |
| Back | 255 | 34.0 |
| Abdomen | 82 | 10.9 |
| **Pubic** | 7 | 0.9 |
| **Groin** | 9 | 1.2 |
| **Buttock** | 55 | 7.3 |
| **Lower extremity** | 90 | 12.0 |
| **Cervical nerve** | 18 | 2.4 |
| **Thoracic nerve** | 41 | 5.5 |
| **Lumbar nerve** | 26 | 3.5 |
| **Sacral nerve** | 9 | 1.2 |
| **Disseminated** | 5 | 0.7 |

**Note:** NLP identified the HZ anatomic location for 751 out of the 800 cases. The percentage was calculated using 751 as the denominator.
