# Supplementary material for: Natural Language Processing Versus Diagnosis Code–Based Methods for Postherpetic Neuralgia Identification: Algorithm Development and Validation
Source: JMIR Med Inform. 2024 Sep 10;12:e57949. doi: 10.2196/57949 (PMC11407135; doi:10.2196/57949)
Supplement: Multimedia Appendix 6 [file medinform-v12-e57949-s006.docx]

**Appendix 6. Reported PHN Rates in Previous Administrative Database Studies**

| **Author** | **PHN duration** | **Year** | **PHN cases** | **Age** | **PHN (%)** |
| --- | --- | --- | --- | --- | --- |
| Opstelten | ≥90 days | 1994–1999 | 22 | All ages | 2.6 |
| Ultsch | ≥90 days | 2004–2009 | 18,160 | All ages | 4.5 |
| Gialloreti | ≥90 days | 2003–2005 | 350 | ≥50 years | 6.2 |
| Schiffner-Rohe | ≥90 days | 2004 | NA | ≥50 years | 6.9 |
| Jih | ≥90 days | 2000–2006 | 2,944 | All ages | 8.6 |
| Gauthier | ≥90 days | 2000–2006 | 415 | ≥50 years | 13.7 |

The selected studies were from Kawai K, Gebremeskel BG, Acosta CJ. Systematic review of incidence and complications of herpes zoster: towards a global perspective. *BMJ Open*. Jun 10 2014;4(6):e004833.

Opstelten W, Mauritz JW, de Wit NJ, et al. Herpes zoster and postherpetic neuralgia: incidence and risk indicators using a general practice research database. Fam Pract 2002;19:471–5.

Ultsch B, Köster I, Reinhold T, et al. Epidemiology and cost of herpes zoster and postherpetic neuralgia in Germany. Eur J Health Econ 2013;14:1015–26.

Gialloreti LE, Merito M, Pezzotti P, et al. Epidemiology and economic burden of herpes zoster and post-herpetic neuralgia in Italy: a retrospective, population-based study. BMC Infect Dis 2010;10:230.

Schiffner-Rohe J, Jow S, Lilie HM, et al. Herpes zoster in Germany. A retrospective analyse of SHL data. MMW Fortschr Med 2010;151(Suppl 4):193–7.

Jih JS, Chen YJ, Lin MW, et al. Epidemiological features and costs of herpes zoster in Taiwan: a national study 2000 to 2006. Acta Derm Venereol 2009;89:612–16.

Gauthier A, Breuer J, Carrington D, et al. Epidemiology and cost of herpes zoster and post-herpetic neuralgia in the United Kingdom. Epidemiol Infect 2009;137:38–47.
